# Robert Killick-Kendrick (1929-2011): *In memoriam*

**DOI:** 10.1051/parasite/2012193290

**Published:** 2012-08-15

**Authors:** J.A. Rioux, I. Landau, R. Houin


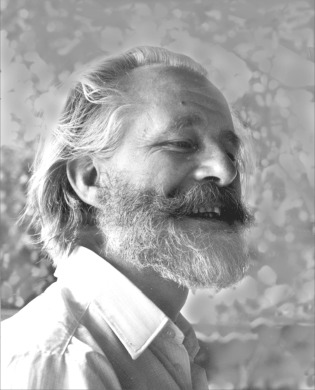


Robert Killick-Kendrick est décédé le 22 octobre 2011 à Sumène, dans ce Pays cévenol où il avait choisi de se retirer après une longue vie de chercheur, initiée à l’âge de 17 ans au Département de biochimie du Ministère britannique de l’agriculture. Huit années au Nigeria, passées à étudier les trypanosomoses humaines et animales, devaient constituer le prologue d’une carrière consacrée à une parasitologie ouverte sur tous les champs de la connaissance.

En 1963, il rejoignait le service du Professeur P.C.C. Garnham à la *London School of Hygiene and Tropical Medicine*. À cette époque, le laboratoire fourmillait de jeunes chercheurs qui, presque tous, devaient poursuivre de remarquables carrières : J. Baker, R. Bray, R. Lainson et J. Shaw. S’y ajoutaient L. Canning de l’*Imperial College* et M. Wery de l’École de Médecine tropicale d’Anvers. Des visiteurs y venaient du monde entier pour effectuer des stages ou s’entretenir de leurs recherches. L’activité était foisonnante et efficace : les chercheurs appelaient leurs collègues dès qu’ils découvraient un élément nouveau ou lorsqu’ils avaient besoin d’une aide technique. Bob était l’âme de ce petit groupe. Toujours disponible, il se passionnait pour les faits exceptionnels et apportait alors le renseignement ou l’aide utile.

À l’époque, le concept de “biodiversité” n’était pas encore à la mode. Mais, à la *London School*, c’était le principe fondateur du laboratoire. On y étudiait non seulement les parasites de l’Homme et de Primates, mais aussi ceux d’autres Mammifères, des Oiseaux, des Reptiles et des Insectes. Les approches en étaient systématique, biologique, immunologique et épidémiologique. Leur étude était facilitée par l’abondance du matériel (frottis de sang, coupes de tissu) : une manne provenant du monde entier.

Le paludisme était l’un des thèmes principaux de recherche et, dans ce domaine, Bob participait activement à toutes les expériences sur les *Plasmodium* de Primates. Son apport personnel en fut considérable. Citons :

1. la description et l’établissement du cycle de deux *Plasmodium* de Rongeurs – *Plasmodium yoelii yoelii* et *P. chabaudi chabaudi* –, espèces devenues de véritables modèles expérimentaux (1966);
2. la description de *Plasmodium yoelii nigeriensis* et de *P. vinckei brucechwatti* chez *Thamnomys rutilans* du Nigeria (1975);
3. la description de deux espèces nouvelles de *Plasmodium* chez des Anomaluridae de Côte d’Ivoire (1973);
4. la production de gamétocytes de *P. yoelii* par les schizontes préérythrocytaires (1968);
5. la première observation de résistance à la chloroquine de *Plasmodium yoelii* (1967);
6. la découverte chez *Plasmodium cynomolgi* d’une forme quiescente, ou “hypnozoïte”, par une équipe de huit chercheurs dont Bray et Garnham. Plus tard, cette forme sera observée chez *P. vivax* et *P. ovale* : une découverte majeure qui permettra d’élucider l’origine des rechutes chez ces deux espèces (1982);
7. la participation à la Mission Bornéo “Recherches des *Plasmodium* de l’Orang-outan”, avec la description de *P. sylvaticum* et la réalisation du cycle expérimental;
8. le chapitre “*Taxonomy, Zoogeography and Evolution*” du livre *Rodent malaria* (co-édité avec W. Peters, 1978) : un ouvrage qui reste encore d’une grande actualité.

C’est aussi pendant cette période qu’il acquit, sous l’impulsion de Garnham, les titres universitaires qui lui permirent de disposer des moyens nécessaires à ses recherches. La longue liste des distinctions qui lui ont été remises témoigne de l’estime dont l’ont gratifiée ses maîtres et ses pairs.

Il nous faut à présent évoquer quelques-uns des temps forts qui ont marqué la seconde vie de Bob, celle du “leishmaniaque” : une activité de tous les instants, menée tant dans l’Ancien que le Nouveau Monde, comme en témoignent les nombreuses missions effectuées à la demande des pays ou des grands organismes internationaux. Toutefois, afin d’éviter un trop long exposé, nous nous contenterons d’évoquer quelques-unes des études de terrain, menées en France méditerranéenne, dans les foyers leishmaniens. Ce faisant, nous espérons restituer l’ambiance de créativité qui fut, durant plusieurs années, la conséquence de cette étroite coopération.

Les premiers contacts de Killick- Kendrick avec Montpellier datent des années 1960. À cette époque, se terminait une longue enquête sur “L’épidémiologie des leishmanioses dans le sud de la France”. Conduite par l’équipe de recherche “Écologie parasitaire” de la Faculté de Médecine, cette opération s’était déroulée dans la Montagne Noire. En 1969, les résultats en étaient restitués sous la forme d’une Monographie INSERM, dont Garnham avait publié une analyse élogieuse. Par la suite, il avait souhaité visiter les différents sites étudiés. Pour ce faire, l’équipe montpelliéraine avait organisé un itinéraire de démonstration hautement évocateur. Bien entendu, Bob était du voyage. S’étaient également joints au groupe A. Chabaud et I. Landau. Le périple nous avait conduits jusqu’à la station océanographique de Banyuls-sur-Mer (Pyrénées-Orientales), petit port catalan où, dans l’immédiat aprèsguerre, Garnham avait terminé sa traversée de la France. Et c’est à l’arrivée à Banyuls que ce dernier avait suggéré à son élève de s’associer à l’équipe de Montpellier. Cette “suggestion” fut le point de départ d’une fructueuse collaboration, d’autant que Garnham allait convaincre plusieurs collaborateurs britanniques de “descendre” dans le Midi méditerranéen.

Quelques années plus tard, la qualité de cette association était confirmée à l’occasion d’un congrès sur l’Écologie des Leishmanioses, organisé par l’Université Montpellier 1 (1974). Autour de Garnham, son président, était regroupé un nombre impressionnant de spécialistes étrangers : N. Ansari, R. Ashford, A. Beliaev, R. Bray, M. Chance, J. Collado, A. Corradetti, P. De Raadt, J. Jadin, E. Jadavian, R. Lainson, D. Le Ray, D. Lewis, W. Lumsden, G. Lupascu, D. Molineux, A. Nadim, W. Peters, V. Safyanova, V. Sergiev, T. Simic, B. Southgate, O. Theodor, S. Yasarol, sans oublier le secrétaire du congrès : R. Killick-Kendrick. Pour certains, le discours d’ouverture de Garnham fut une véritable révélation. D’ailleurs, le titre en était prémonitoire : *Global ecology of the leishmaniases: introductory rermarks*. Pour ce maître à penser, “l’écologie parasitaire” ne manquerait pas de s’affirmer comme une discipline heuristique. Et c’est effectivement ce qu’il advint : l’écologie des leishmanioses fut l’un des thèmes de recherches essentiels pour plusieurs équipes internationales.

Mais revenons “au sens” qu’avait donné Bob à son parcours de chercheur. Certes, il adorait le travail de paillasse : il y manifestait une remarquable efficacité méthodologique et technique. Mais ses qualités ne s’épanouissaient vraiment qu’*in natura*, au sein des foyers d’infection. Pour résoudre les énigmes qui le passionnaient, il n’hésitait pas à passer des nuits à disséquer ses captures et, dans le même temps, engageait de longues discussions sur l’épistémologie des “complexes pathogènes”. À ces occasions, il faisait volontiers référence aux disciplines écologiques de base que sont la systématique, l’éthologie et la biogéographie. Oui ! Bob fut vraiment un authentique éco-épidémiologiste.

Concrètement, notre collaboration débutait sur le versant méridional du massif cévenol de l’Oiselette, entre Ganges et Le Vigan. Dans cette zone, plusieurs cas de leishmanioses viscérales infantiles avaient été dépistés quelques années auparavant. La commune de Roquedur, située à mi-pente, était choisie comme point d’ancrage de l’équipe. Un “laboratoire de campagne” était installé dans une maison traditionnelle. Les pièges lumineux, les pièges adhésifs, les capturateurs à bouche, les appareils d’optique, les instruments de dissection, les milieux de culture, les chiens leishmaniens et les phlébotomaires, tout était en état de marche. L’équipe franco-britannique, composée d’une vingtaine de chercheurs, était opérationnelle : l’enquête éco-épidémiologique pouvait débuter. Parmi les nombreux sujets développés dans la station, nous en présenterons trois, parmi les plus représentatifs :

1. Le premier concerne l’infestation expérimentale par *L. infantum* du chien et de *P. ariasi*. Elle portait sur deux chiots naïfs mis en contact, sous phlébotomaire, avec des insectes femelles préalablement infestées. Après quelques semaines, l’un des animaux succombait après un épisode sidérant. Le second, asymptomatique durant plus d’un an, réalisait *ex abrupto* une émission anale de sang rouge, symptôme pathognomonique d’une forme viscéro-cutanée chronique. Dans les deux cas, la transmission par *P. ariasi* était démontrée.
2. Le deuxième sujet se rapporte au développement de *L. infantum* chez *P. ariasi*. Imprégné des idées de Garnham, Bob s’était intéressé aux transformations des *Leishmania* chez le vecteur. Plusieurs raisons, d’ordre pratique, favorisaient ces recherches : a) en Cévennes, *P. ariasi* pouvait être massivement infesté sur un chien leishmanien; b) à cette époque, il était possible d’effectuer ce type d’études sans contraintes médico-administratives excessives. Quoi qu’il en soit, les résultats en étaient spectaculaires. Bob retrouvait les formes intravectorielles observées au Brésil, qu’il s’agisse des traditionnels pro-mastigotes en division, des méta-promastigotes, des para-, des sphéro- et des micro-mastigotes, libres ou fixés sur la paroi intestinale. Au surplus, des coupes minces permettaient de détecter les hémi-desmosomes, ces structures sous-épithéliales énigmatiques, développées au contact du parasite.
3. Le troisième thème porte sur les distances de vol des Phlébotomes. Exception faite des travaux russes sur *P. papatasi*, il était traditionnel de considérer les déplacements des Phlébotomes comme de simples sauts, de faible amplitude. Dans ces conditions, les vecteurs ne pouvaient assurer le transport du parasite sur de longues distances : seuls les mammifères-réservoirs en étaient capables. Or, dans l’enquête antérieure en Montagne Noire, des passages directionnels de femelles de *P. ariasi* au-dessus des Chênes verts avaient été observés. À Roquedur, la confirmation du phénomène était apportée grâce à la méthode de recapture après marquage aux poudres fluorescentes. Sans nous étendre plus longuement sur l’opération ellemême, très lourde au demeurant, on constatait que certains *P. ariasi*, relâchés aux environs de Roquedur (vallée de l’Hérault, alt. 250 m), s’étaient déplacés sur près de cinq kilomètres. En trois jours, plusieurs femelles, dont certaines gorgées, avaient franchi un col situé à 600 m d’altitude, pour redescendre sur le versant opposé (vallée de l’Arre, alt. 300 m). Un résultat riche d’enseignements : les contaminations humaines (ou animales), pour lesquelles aucun chien leishmanien n’avait été dépisté aux environs, pouvaient s’expliquer par les grands déplacements de vecteurs infestés.

Au demeurant, nous ne saurions terminer cet *in memoriam*, sans rendre hommage à Mireille Killick- Kendrick, son épouse dévouée et sa collaboratrice de tous les instants. Dans les laboratoires d’Ascot (Grande-Bretagne) et de Sumène (France), elle avait établi plusieurs colonies de Phlébotomes et, récemment, avait participé à la mise au point d’un collier imprégné de pyréthrinoïdes, technique utilisée avec succès dans la prévention de la leishmaniose canine.

Une nouvelle fois, Bob, nous te redisons notre très amicale sympathie, sans oublier l’admiration et la reconnaissance que n’ont pas manqué de te manifester les nombreux élèves que tu as formés et les collègues qui t’ont connu.

